# Improved representation of atmospheric dynamics in CMIP6 models removes climate sensitivity dependence on Hadley cell climatological extent

**DOI:** 10.1002/asl.1073

**Published:** 2021-11-02

**Authors:** Bithi De, George Tselioudis, Lorenzo M. Polvani

**Affiliations:** ^1^ NASA Goddard Institute of Space Studies (GISS) New York New York USA; ^2^ Department of Applied Physics and Applied Mathematics Columbia University New York New York USA; ^3^ Department of Earth and Environmental Sciences, and Lamont‐Doherty Earth Observatory Columbia University New York USA

**Keywords:** Cloud Radiative Effects, CMIP6, Equilibrium Climate Sensitivity, Hadley cell

## Abstract

The persistent inter‐model spread in the response of global‐mean surface temperature to increased CO_2_ (known as the “Equilibrium Climate Sensitivity,” or “ECS”) is a crucial problem across model generations. This work examines the influence of the models' present‐day atmospheric circulation climatologies, and the accompanying climatological cloud radiative effects, in explaining that spread. We analyze the Coupled Model Intercomparison Project Phase 6 (CMIP6) models and find that they simulate a more poleward, and thus more realistic, edge of the Hadley cell (HC) in the Southern Hemisphere than the CMIP5 models, although the climatological shortwave cloud radiative effects are similar in the two generations of models. A few CMIP5 models with extreme equatorward biases in the HC edge exhibited high ECS due to strong Southern midlatitude shortwave cloud radiative warming in response to climate change, suggesting an ECS dependence on HC position. We find that such constraint no longer holds for the CMIP6 models, due to the absence of models with extreme HC climatologies. In spite of this, however, the CMIP6 models show an increased spread in ECS, with more models in the high ECS range. In addition, an improved representation of the climatological jet dynamics does not lead to a new emergent constraint in the CMIP6 models either.

## Graphical abstract

Coupled Model Intercomparison Project Phase 6 (CMIP6) models simulate more poleward, and thus more realistic, current climate Hadley cell (HC edge) extent than their CMIP5 counterparts in austral summer (a). The improved Hadley Cell climatology does not explain the increased CMIP6 spread in equilibrium climate sensitivity (ECS) values (b). A subset of CMIP5 models with extreme equatorward biases in the HC tended to have higher ECS values due to anomalous Southern midlatitude shortwave cloud radiative warming, producing an ECS dependence on HC position. No such constraint holds in CMIP6.

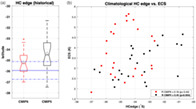

**Abbreviations**
CMIP6: Coupled Model Intercomparison Project Phase 6
CRE: Cloud Radiative Effects
ECS: Equilibrium Climate Sensitivity
HC: Hadley cell

## 1 INTRODUCTION

The poleward shift in the Hadley cell (HC), and of the midlatitude eddy‐driven jet, have been identified as a robust dynamical signal to increasing CO_2_ across the latest generation of climate models (Curtis et al., 2020; Grise and Davis, 2020, and references therein). The poleward expansion of the tropics has potentially important consequences on the subtropical climate such as pushing the dry zones poleward (e.g. Lu et al., 2007; Scheff and Frierson, 2012; Chemke and Polvani, 2019). Such changes in the large‐scale circulation may impact clouds and the associated cloud radiative effects (CRE) that are responsible for most of the uncertainty in the climate change projections (Grise and Polvani, 2014b; Kay et al., 2014; Bony et al., 2015; Ceppi and Hartmann, 2015; Wall and Hartmann, 2015; Lipat et al., 2017, and references therein). In an observational study, Tselioudis et al. (2016) linked the midlatitude clouds and CRE with the poleward expansion of the HC, rather than with the poleward shift of the midlatitude jet. Other studies have shown that models have not been able to simulate the observed sensitivity of clouds and radiation to dynamical shifts of the midlatitude jet (Grise and Polvani, 2014b; Grise and Medeiros, 2016). Quantifying the source of uncertainty in the model radiative response to dynamical shifts is necessary for a better understanding of the future climate projection.

Lipat et al. (2017) found that in the Coupled Model Intercomparison Project Phase 5 (CMIP5) climate warming simulations, models with excessively narrow climatological HC produced a stronger Short Wave (SW) CRE warming over the Southern lower midlatitudes region (28^°^–48^*°*^S), and this contributed to higher model Equilibrium Climate Sensitivity (ECS) values; they suggested the possibility of an emergent constraint based on the climatological position of the HC edge in the Southern Hemisphere. They hypothesized that improved representation of atmospheric dynamics in later model generations would result in smaller future warming at Southern midlatitudes, and would thus favor lower model ECS values. In contrast, Zelinka et al. (2020) recently reported overall increased ECS values for the CMIP6 models; they also showed that it can be attributed, to a large extent, to higher SWCRE warming over that same Southern midlatitudes region. Furthermore, Schlund et al. (2020) reported that the climatological HC position no longer offers an emergent constraint on ECS in the CMIP6 models, however, the underlying reasons remain unexplained. The recent study of Curtis et al. (2020) found a more poleward climatological jet position in CMIP6 than in CMIP5 models, and suggested this contributes to a muted poleward jet shift averaged from May to October in CMIP6 climate warming simulations. However, the correlation between jet position and jet shift was found to be much smaller (0.42) for the November to April average of interest in this paper (the standard December–January–February mean was not reported). Furthermore, such relationship between the models' equatorward jet bias and their jet shift under CO_2_ forcing, earlier proposed by Kidston and Gerber (2010), was shown to be inconsistent with the fluctuation‐dissipation theorem by Simpson and Polvani (2016) and is therefore unclear why a smaller climatological bias would cause a smaller response. Nonetheless, it remains important to explore whether and how the changes in the representation of atmospheric dynamics affect the associated CRE feedbacks in the CMIP6 model ensemble. The present study aims to understand why the emergent constraint, as hypothesized by Lipat et al. (2017), does not hold by the newer generation of climate models (Schlund et al., 2020) with an improved representation of the simulated dynamics. Specifically, we assess the systematic biases in dynamically driven SWCRE feedbacks and their relationship with ECS across two generations of the models. Building on this very idea, we also explore if the improved jet dynamics and associated SWCRE feedbacks lead to a new emergent constraint in the CMIP6 models.

In the present study, we examine the most recent CMIP6 model simulations to test whether changes in the representation of climatological HC extent in the Southern Hemisphere affect those models' equilibrium climate sensitivities. The objective of the study is pursued in two analysis steps. First, we map and evaluate the representation of the HC and CRE in CMIP6 models, and contrast those to their CMIP5 counterparts. To do this, we compare the climatological HC in the models with reanalyses, and the climatological SWCRE in the models (in terms of intensity) with satellite observations. Second, we diagnose the link between the simulated HC extent and the SWCRE at Southern midlatitudes, and the spread in ECS across model generations. We also diagnose the relationship between the simulated jet and the SWCRE over Southern midlatitudes. The paper is organized as follows. Section 2 details the models, observational data and methodology used in this study. Section 3 discusses the dynamical representation across model generations, and the associated impacts on the dynamical and climate sensitivities. Section 4 concludes the paper.

## 2 DATA AND METHODS

### 2.1 Data

We analyze the output from 24 CMIP6 models (Eyring et al., 2016) and 24 CMIP5 models (Taylor et al., 2012), as listed in Table S1. We use the monthly mean output of the dynamical variables and SWCRE fluxes from the historical runs, the preindustrial (PI)‐control runs, and the abrupt 4*X*CO_2_ runs for each model. The fully coupled historical runs are used to compare the simulations with the reanalyses and observations over the satellite era. In order to avoid weighting biases among models, we analyze only the first ensemble simulations in each model. We select the models based on the availability of dynamical and radiative output, and equilibrium climate sensitivity (ECS) values. The ECS value for each model is obtained from Zelinka et al. (2020). We use the PI‐control runs to examine the climatological cloud‐dynamics interaction. The long integration period of the PI control runs is useful for obtaining a statistically robust understanding of each model's climatology. For a subset of models (based on availability), we analyze the climatology from AMIP simulations as well. The AMIP simulations, prescribed with observed sea surface temperature (SST), can provide insight whether a biased SST distribution in the coupled simulations contributes to the biased spread in circulation metrics.

We use monthly‐mean zonal wind and meridional wind data from the European Centre for Medium‐Range Weather Forecasts Interim Reanalysis (ERA‐Interim) (Dee et al. 2011) to calculate the HC location. The observational SW radiative flux data is monthly top‐of‐the‐atmosphere (TOA) radiative fluxes derived from the International Satellite Cloud Climatology Project (ISCCP‐H) (Young et al., 2018) during 1984–2016 period.

### 2.2 Methods

One dynamical metric and one radiative metric, used in this study, are defined as follows:

1. The poleward edge of HC (*ϕ*_*ψ*500_) is calculated as the latitude where the mid‐tropospheric (500 hPa) meridional mass stream function (*ψ*_500_) first changes sign poleward of its maximum absolute value in the tropics, as in Chemke and Polvani (2019). We calculate this metric (defined as PSI500) using the tropical‐width diagnostics code package (TropD; Adam et al., 2018).
2. Shortwave cloud radiative effect (SWCRE) is computed as TOA upwelling clear sky SW radiation (model output variable ‘rsutcs') minus TOA upwelling all‐sky SW radiation (‘rsut’) as in Lipat et al. (2017).

Following Lipat et al. (2017), we here focus on the Southern Hemisphere (SH) during the austral summer (DJF) when the insolation and associated SWCRE are at maximum, but we also discuss the seasonality by considering austral winter (JJA). We focus on the zonal mean SWCRE over Southern lower midlatitude region (hereafter, LML), defined as the latitude band from 30^°^ to 50^°^S that shows the maximum change in SWCRE (Zelinka et al., 2020). The SWCRE response to increased CO_2_ (as shown in Figures 2a and 3b) is diagnosed as the difference between the abrupt 4*X*CO_2_ run (with first 50 years removed) and the PI‐control run climatology, following Lipat et al. (2017). In addition, to avoid the fast adjustment of atmospheric circulation response to direct radiative effect of CO_2_, we compute temperature‐mediated SWCRE response (as shown in Figure S2); where we regress detrended timeseries of SWCRE anomalies onto detrended timeseries of global mean surface temperature (TS) anomalies and the responses are calculated as the difference between the 4*X*CO_2_ climatology over full available simulation period (150 years) compared to the PI‐control climatology. From the regression, TS‐SWCRE is defined as the rate of change in SWCRE over SH LML due to 1 K change in global surface temperature to assess the relationship between SWCRE changes associated with surface warming and ECS.

We use 4*X*CO_2_ runs instead of the Representative Concentration Pathway (RCP) runs primarily to obtain a robust signal with a large forcing (Grise and Polvani, 2014a). Finally, as part of the DECK, the 4*X*CO_2_ runs are a standard experiment that will be included in all future CMIP phases (Eyring et al., 2016; Grise and Davis, 2020). In order to examine the interannual covarability using PI‐control, following Lipat et al. (2017), the detrended SWCRE timeseries are regressed onto detrended *ϕ*_*ψ*500_ timeseries. We define the HC‐SWCRE as the SWCRE changes due to 1^°^ poleward shift in HC edge latitude. Statistical significance is calculated using two‐tailed Student's *t* test.

## 3 RESULTS

### 3.1 Dynamical and radiative representation in the CMIP6 climatology

**FIGURE 1:**
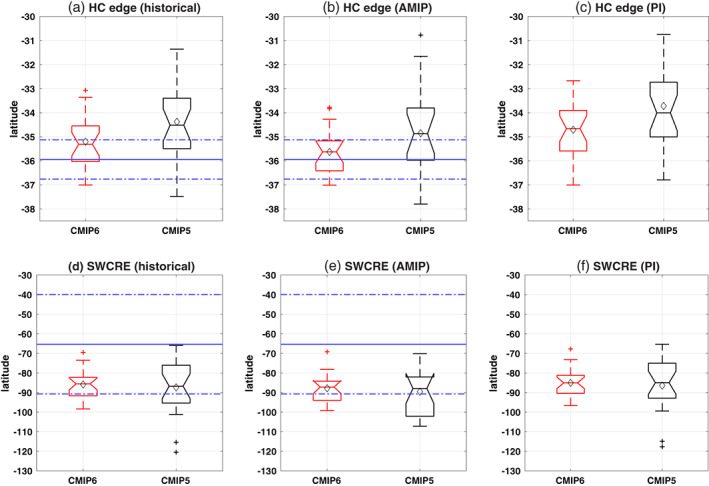
Magnitude of the historical climatology (over the period of 1979–2014) in SH DJF are shown for (a) Hadley cell edge and (d) SWCRE over SH LML region, respectively for CMIP6 and CMIP5 models as listed in the Table S1. The boxes represent the 25–75 percentiles of the inter‐model ranges, notch represents the median, black diamond represents the multi‐model mean value and the whiskers show the outlier models, respectively, in each CMIP group. The blue dotted lines denote the observational variability, defined as 1 *SD* (over the same time period) and the blue solid line denotes the mean observed value for the corresponding variable. CMIP6 and CMIP5 ensemble are shown in red and black boxes, respectively. (b) and (e) are the same as (a) and (d) but for the subset of models with available AMIP runs. (c) and (f) are the same as (a) and (d) but for the full integration period of PI‐control runs

First, we diagnose the historical climatology of the CMIP models during austral summer and compare with that of the reanalysis and observations to identify any improvement across model generations. In Figure 1a,d, the climatological distribution of HC edge and SWCRE over the LML region, across model generations, are shown with box‐and‐whisker plots, while the observed mean and range for the same variables are shown with blue lines. A wide inter‐model spread in climatological HC edge and SWCRE is seen in both CMIP6 and CMIP5 models (Figure 1a,d). However, the CMIP5 distribution is considerably larger, as it includes a tail of extreme models with narrow HC and low SWCRE values; the CMIP6 model distribution is more tightly confined.

A comparison with reanalysis reveals that, while CMIP5 models have the HC edge skewed towards the equator, the CMIP6 models have that feature shifted towards the poles and thus, falling more within the observed range (shown as dotted blue lines in Figure 1a). The intermodel spread in climatological HC edges in CMIP6 models is clearly reduced, with more realistic magnitudes for both mean (shown as a black diamond) and median (shown as a notch and bar) values than that in the CMIP5 historical runs. The spread in the distribution of climatological HC edges, using CMIP6 AMIP runs, further reduces compared to that in the historical runs; and therefore, becomes more realistic (Figure 1b). Comparison of the modeled LML SWCRE with satellite observations, however, shows that the mean and median values remain comparable between the two generations of models, and fall within the observed variability (Fig. 1d,e). CMIP6 models have a narrower distribution for climatological SWCRE; therefore, more models are within the observed boundaries than the CMIP5. Note that, in historical CMIP5 runs, there are two models that exhibit an extreme SWCRE while no such behaviors exist in the AMIP runs. Similar results are also seen in the PI‐control runs (Fig. 1c,f), except the CMIP5 HC edge is found to be more towards the equator with PI forcings. We also examine that different subset of models, for historical and AMIP runs, do not really affect our main results (not shown). During JJA, the intermodel spread in HC and SWCRE reduces in the CMIP6 models; however, the mean and median remains comparable across both generations (Figure S1).

Overall, we conclude that the CMIP6 models simulate an improved representation of a major atmospheric dynamical cell in the Southern Hemisphere (SH) summer, since they correct to a large extent the equatorward bias of the CMIP5 HC edge. Our analysis shows that this bias is primarily due to a subset of CMIP5 models that have excessively narrow Hadley cells, or a strong negative bias of SWCRE over SH LML (consistent with Bock et al., 2020), well beyond those found in the CMIP6 historical and PI‐control ensemble. We assess that a statistically significant correlation, −0.41 for the CMIP6 and −0.47 for the CMIP5 with 95% confidence level, exists between climatological HC edge and SWCRE over the LML primarily due to the presence of biased models with narrower/wider HC edge or extreme SWCRE values. If we remove these extremely biased models the relationship does not exist anymore (not shown). Interestingly, such relationship, between climatological HC edge and SWCRE, does not exist in the subset of models available for AMIP runs (not shown). This indicates possibility of a biased SST distribution in the CMIP5 coupled runs led to a systematic bias in dynamically driven CRE feedbacks and the associated higher ECS values. Next, we explore the influence of the models' control climatology on the models' future warming projection. In order to better compare with Lipat et al. (2017), the PI control climatology is used for rest of the manuscript.

### 3.2 Influence of climatology on the radiative feedback and climate sensitivity

**FIGURE 2:**
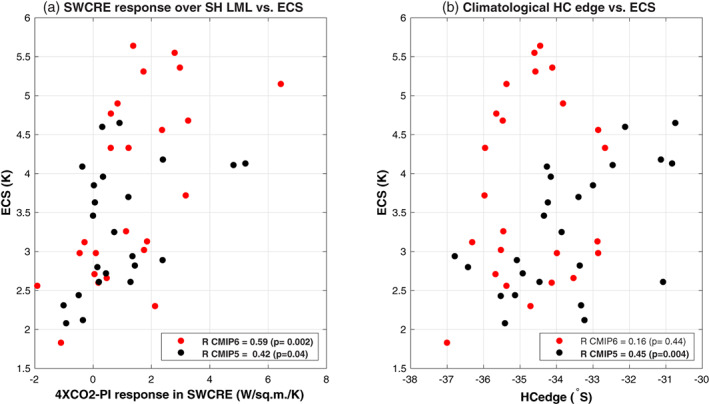
Relationship between the intermodel spread in the (a) response in SWCRE from the PI‐control climatology to the 4*X*CO_2_ climatology over SH LML (lower midlalitudes defined over 30^°^–50^°^S) for DJF and ECS values for each model. The SWCRE response is normalized by corresponding ECS values. (b) Climatological DJF SH Hadley cell latitude from PI‐control runs and ECS values. The individual models from CMIP6 ensemble are shown in red dots and from the CMIP5 models are shown in black dots. The correlation coefficient (R) and the statistical significance (*p*) corresponding to each group are shown in the legends. The significant correlations have been bolded

We here examine now how the representation of Hadley cell climatology affects the simulated LML SWCRE response over the Southern midlatitudes and, potentially, the ECS across model generations. In Figure 2a, we present the relationship between 4*X*CO_2_‐PI difference in SWCRE and ECS, and Figure 2b shows the relationship between the climatological HC edge and ECS. First, a significant positive correlation can be seen between the LML SWCRE response and ECS, in both CMIP5 and CMIP6 models (Figure 2a): this suggests that this regional SWCRE response contribute to a larger global mean surface warming, in line with the previous findings (Lipat et al., 2017; Zelinka et al., 2020). However, as one can see in Figure 2b, while in CMIP5 models ECS is correlated with the climatological position of the HC edge (*R*~0.45), this is not the case in the CMIP6 model ensemble (*R*~0.16). This confirms the recent results of Schlund et al. (2020), who concluded that the climatological HC edge is not an emergent constraint on ECS in the CMIP6 models, as suggested by Lipat et al. (2017) using the CMIP5 models. In addition, in order to avoid influence from fast adjustment of atmospheric circulation response to direct radiative effect of increased CO_2_, we further examine the relationship between SWCRE responses associated with global mean surface temperature anomalies (TS‐SWCRE, see Section 2.2) and ECS values (Figure S2). A statistically significant positive correlation between LML TS‐SWCRE and ECS suggests a change in SWCRE response associated with rate of change in surface temperature affects the higher ECS values only in the CMIP6 models.

**FIGURE 3:**
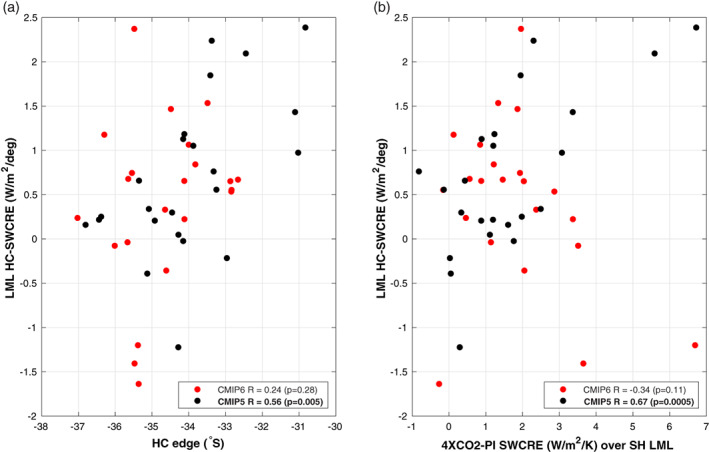
Relationship between LML HC‐SWCRE (see Section 2.2) with (a) climatological DJF SH Hadley cell edge latitude and (B) LML (defined as 30 − 50^°^S) averaged response of the SWCRE to increase CO_2_ (as the 4*X*CO_2_‐PI SWCRE difference). The SWCRE response is normalized by corresponding ECS values. The significant correlations have been bolded

To better understand this difference in behavior between the two model generations, Figure 3A shows the relationship between climatological HC edge position in the PI control runs and the Southern Hemisphere LML HC‐SWCRE relationship (see Section 2.2), for both the CMIP5 and the CMIP6 models. That plot demonstrates that the CMIP5 ensemble models with narrow Hadley cells simulate a strong radiative warming/cooling of the LML when the cell expands/contracts interannually, whereas the CMIP6 ensemble the LML warming/cooling accompanying HC expansion/contraction is largely independent of the climatological HC position. Furthermore, as shown in Figure 3b, the 0.67 correlation between the response of the SWCRE to CO_2_ quadrupling (on the abscissa) and the LML HC‐SWCRE (on the ordinate) seen in the CMIP5 models, is not present in the CMIP6 models.

It is important to note that the existence of a strong correlation between the HC edge climatological latitude and the HC‐SWCRE in the CMIP5 models (Figure 3a) is due to the presence of the four extreme models with unrealistically narrow Hadley cells. Removing those extreme models greatly reduces the significance of the correlation. The results in Figure 3 indicate that the emergent constraint relating to the edge of the HC, proposed in Lipat et al. (2017) on the basis of CMIP5 models, was caused primarily by a subset of extremely biased models, and the improved simulation of the HC in the CMIP6 ensemble made that constraint vanish. However, that improvement did not result in the ensemble ECS shifting towards a lower value, as hypothesized by Lipat et al. (2017). These new findings, therefore, point to cloud microphysics as a more likely possible cause for the enhanced ECS in the CMIP6 models (Zelinka et al., 2020). Moreover, due to lack of relationship between the SWCRE associated with a Hadley cell shift and the SWCRE associated with the climate change response in CMIP6 models (Figure 3b), we examine whether any significant differences occur in the rapid, transient response of the models to the greenhouse forcing (Grise and Polvani, 2014b, and references therein). Therefore, we assess the relationship between the transient SWCRE response to climate change in the SH midlatitudes, averaged over first 10 years and 20 years of the abrupt 4*X*CO_2_ runs and HC‐SWCRE derived from the interannual variability in the control (not shown). No significant relationship in the CMIP6 models indicate that the thermodynamical changes are not masking the fast adjustment in dynamically driven clouds radiative forcing.

## 4 DISCUSSION

We have carried out a systematic comparison of simulated Hadley cell extent, CRE, and climate sensitivity in CMIP6 and CMIP5 model ensembles. The CMIP6 models simulate large‐scale atmospheric dynamics in the Southern Hemisphere that is closer to the observations than the CMIP5 models. Specifically, the CMIP6 models exhibit a more realistic poleward climatological mean state for the HC. We find that an improved representation of the climatological HC extent in the CMIP6 models does not lead to weaker Southern midlatitude SWCRE warming, and to lower ECS values as hypothesized by Lipat et al. (2017). Despite the absence of models with an excessively narrow climatological HC, the CMIP6 ensemble shows even larger Southern midlatitude SWCRE warming than CMIP5, resulting potentially from thermodynamical or microphysical processes, as suggested in Zelinka et al. (2020). In addition, our study suggests that a biased SST distribution in the CMIP coupled runs may be linked with systematic cloud‐dynamical biases in the CMIP5 models and therefore, with the associated emergent constraints as found by Lipat et al. (2017).

We have also extended our analysis to examine the climatological representation of the eddy driven jets, and the influence of jet dynamics on the simulated LML SWCRE and model ensemble ECS. Figure S3 shows the historical climatological distribution of jet latitudes across model generations, and the observed variability range for the same. A wide inter model spread in jet position is seen in both CMIP5 and CMIP6. As for the HC, the climatological jets in the CMIP6 have shifted towards the pole, and thus fall better within the observed variability range compared to that for CMIP5. We find a persistent correlation between the climatological HC edge and jet location across the model generations during DJF (Figure S3B). The relationship indicates that the models with a wider HC (i.e. a poleward HC edge) simulate a more poleward jet latitude. Previously, a strong interannual correlation in DJF between the Southern Hemisphere eddy‐driven jet latitude and Hadley cell edge was reported in CMIP3 models (Kang and Polvani, 2011) and in CMIP5 models (Waugh et al., 2018). Overall, we find that the CMIP6 models simulate an improved representation of the major atmospheric dynamical cells in the Southern Hemisphere (SH), in terms of both HC edge and jet latitudes. However, unlike the HC, the climatological jet‐latitude does not seem to influence ECS values, in either of the CMIP5 or CMIP6 (not shown). Relationship between SWCRE and latitude of the jet in the PI control runs (similar to Figure 3) does not affect the response of SWCRE to increased CO_2_, and thus ECS values. We conclude, therefore, that an improved representation of the climatological jet dynamics in the CMIP6 models also does not lead to a new emergent constraint on ECS (consistent with Grise and Polvani, 2014b).

Finally, we note that in a recent paper Curtis et al. (2020) claim that increased horizontal grid resolution in many CMIP6 models is the key to a poleward mean state and a muted shift in the jets. Chemke and Polvani (2019) highlighted the key role of static stability in modulating the HC response to increased CO_2_ forcing. The underlying reasons that lead to a more realistic simulation of large‐scale dynamics in the CMIP6 ensemble are likely very complex, and lie well beyond the scope of this paper, which is focused on the emergent constraint proposed by Lipat et al. (2017). It is also important to mention that we have here focused only on the zonal mean circulation in the SH, because of its possible linkage to ECS. However, further analysis of the regional responses, and of the NH circulation, would also necessary for a better understanding of the climate change projection across model generations.

## AUTHOR CONTRIBUTIONS

Bithi De: Conceptulization; analysis and writing; **George Tselioudis:** Conceptualization; funding acquisition; resources; supervision; writing – review and editing. **Lorenzo Polvani:** Conceptualization; supervision; writing – review and editing. Bithi De designed the study along with GT; performed the analysis and wrote the manuscript (followed by edits from GT and LMP).

## CONFLICT OF INTEREST

The authors declare no conflict of interests.

## Supporting information


**Appendix** **S1:** Supplementary Information
