# Supplementary material for: Improved representation of atmospheric dynamics in CMIP6 models removes climate sensitivity dependence on Hadley cell climatological extent
Source: Atmos Sci Lett. 2021 Nov 2;23(3):e1073. doi: 10.1002/asl.1073 (PMC9285496; doi:10.1002/asl.1073)
Supplement: Supplementary file 1 — Appendix S1: Supplementary Information [file ASL2-23-0-s001.docx]

*Supporting Information for*

**Improved representation of atmospheric dynamics in CMIP6 models removes climate sensitivity dependence on Hadley Cell climatological extent**

Bithi De^1,2^, George Tselioudis^1^ and Lorenzo M. Polvani^2,3^

1. NASA Goddard Institute for Space Studies, New York, NY, USA5
2. Department of Applied Physics and Applied Mathematics, Columbia University, New York, NY, USA
3. Lamont-Doherty Earth Observatory, Columbia University, Palisades, NY, USA

**Contents of this file**

Tables S1

Figures S1 to S3

|  | **CMIP6 models** | **ECS (CMIP6)** | **CMIP5 models** | **ECS (CMIP5)** |
| --- | --- | --- | --- | --- |
| **1** | BCC-CSM2-MR | 3.02 | **ACCESS1-0** | 3.85 |
| **2** | BCC-ESM1 | 3.26 | BCC-CSM1-1 | 2.82 |
| **3** | CAMS-CSM1-0 | 2.29 | BCC-CSM1-1-M | 2.89 |
| **4** | CESM2 | 5.15 | CCSM4 | 2.94 |
| **5** | CESM2-WACCM | 4.68 | CNRM-CM5 | 3.25 |
| **6** | CNRM-CM6-1 | 4.90 | CSIRO-Mk3-6-0 | 4.09 |
| **7** | CNRM-CM6-1-HR | 4.33 | CanESM2 | 3.7 |
| **8** | CanESM5 | 5.64 | FGOALS-s2 * | 4.18 |
| **9** | E3SM-1-0 | 5.31 | GISS-E2-H | 2.31 |
| **10** | EC-Earth3-Veg | 4.33 | **GISS-E2-R** | 2.12 |
| **11** | FGOALS-f3-L | 2.98 | HadGEM2-ES | 4.6 |
| **12** | GISS-E2-1-G | 2.71 | IPSL-CM5A-LR* | 4.13 |
| **13** | GISS-E2-1-H | 3.12 | IPSL-CM5B-LR* | 2.61 |
| **14** | HadGEM3-GC31-LL | 5.55 | **IPSL-CM5A-MR** | 4.11 |
| **15** | INM-CM4-8 | 1.83 | MIROC-ESM * | 4.65 |
| **16** | IPSL-CM6A-LR | 4.56 | MIROC5 | 2.72 |
| **17** | MIROC6 | 2.6 | MPI-ESM-LR | 3.63 |
| **18** | MIROC-ES2L | 2.66 | MPI-ESM-P | 3.46 |
| **19** | MPI-ESM1-2-HR | 2.98 | MRI-CGCM3 | 2.61 |
| **20** | MRI-ESM2-0 | 3.13 | NorESM1-M | 2.8 |
| **21** | NESM3 | 4.77 | INMCM4 | 2.08 |
| **22** | NorESM2-LM | 2.56 | GFDL-CM3 | 3.96 |
| **23** | SAM0-UNICON | 3.72 | GFDL-ESM2G | 2.43 |
| **24** | UKESM1-0-LL | 5.36 | GFDL-ESM2M | 2.44 |

Table S1. List of CMIP6 and CMIP5 models and corresponding ECS values (in K) used in this study. The ECS values are reproduced from Table S1 and S2 of Zelinka 2020. The CMIP5 models not used in Lipat et al. 2017 are bolded. The CMIP5 models with extremely equatorward HC edge have been starred, Following Lipat et al. 2017. 23 models (except CNRM-CM6-1-HR) are used for CMIP6 HC edge analysis using historical runs to produce Fig. 1A. The subset of models present in AMIP runs are highlighted in blue.

**Figure S1**. Similar to Fig.1A and Fig.1D but for SH JJA.

**FIGURE S2.** Relationship between the intermodel spread in TS-SWCRE over southern LML (lower midlalitudes defined over 30°S-50°S) for DJF and ECS values for each model. The individual models from CMIP6 ensemble are shown in red dots and from the CMIP5 models are shown in black dots. The correlation coefficient (R) and the statistical significance (p) corresponding to each group are shown in the legends. The significant correlation has been bolded.

**Figure S3**. (A) Similar to Fig.1A but for eddy driven jet latitudes. (B) relation between climatological jet latitude vs the climatological HC edge in SH DJF. The individual models from CMIP6 ensemble are shown in red dots and from the CMIP5 models are shown in black dots. The correlation coefficient and the statistical significance corresponding to each group are shown in the legends. The significant correlations have been bolded.
